# Research Progress on the Microstructure Evolution Mechanisms of Al-Mg Alloys by Severe Plastic Deformation

**DOI:** 10.3390/ma17174235

**Published:** 2024-08-27

**Authors:** Chang-Rong Song, Si-Yu Zhang, Lin Liu, Hong-Yu Yang, Jie Kang, Jia Meng, Chang-Jie Luo, Cheng-Gang Wang, Kuang Cao, Jian Qiao, Shi-Li Shu, Ming Zhu, Feng Qiu, Qi-Chuan Jiang

**Affiliations:** 1 Key Laboratory of Automobile Materials, Ministry of Education and Department of Materials Science and Engineering, Jilin University, Renmin Street No. 5988, Changchun 130025, China; scr980813@163.com (C.-R.S.); syzhang1622@mails.jlu.edu.cn (S.-Y.Z.); yanghongyu2021@jlu.edu.cn (H.-Y.Y.); jqc@jlu.edu.cn (Q.-C.J.); 2 United Automotive Electronic Systems Limited Company, Rongqiao Street No. 555, Pudong New Area, Shanghai 201206, China; 3 Jilin Liyuan Precision Manufacturing Co., Ltd., No. 5729, Xi’ning Road, Economic Development Zone, Liaoyuan 136299, China; xinzheng@liyuanjingzhi.net.cn; 4 Department and Test Center, FAW—Volkswagen Automotive Co., Ltd., Changchun 130011, China; jia.meng@faw-vw.com; 5 Cansinga Technology Co., Ltd., Building D, Central Avenue, Bao’an District, Shenzhen 518101, China; cj.luo@cansinga.com; 6 Technology Research and Development Casting & Forging Research Institute, FAW Foundry Co., Ltd., Crossing of Hexie Street & Bingwu Road Automotive Industry Development Zone, Changchun 130013, China; 18704456086@163.com; 7 Jiangsu Dalishen Aluminum Industry Co., Ltd., No. 8 Shengchang West Road, Economic Development Zone, Zhenjiang 212314, China; sales@dls-group.com.cn; 8 School of Mechatronic Engineering and Automation, Foshan University, No. 33 Guangyun Road, Nanhai District, Foshan 528231, China; qiaojj99065@163.com; 9 School of Mechanical and Aerospace Engineering, Jilin University, Renmin Street No. 5988, Changchun 130025, China; shushili@jlu.edu.cn; 10 Zhenjiang Xianfeng Automotive Parts Co., Ltd., Dantu High Tech Industrial Park, Zhenjiang 212000, China; ming.zhu@xfelectronic.cn

**Keywords:** Al-Mg alloys, SPD, bimodal microstructure, nanotwin microstructure, nanoparticles, high strength and high toughness

## Abstract

Al-Mg alloys are widely used as important engineering structural materials in aerospace engineering, transportation systems, and structural constructions due to their low density, high specific strength, corrosion resistance, welding capability, fatigue strength, and cost-effectiveness. However, the conventional Al-Mg alloys can no longer fully satisfy the demands of practical production due to difficulties caused by many defects. The high strength of Al-Mg alloys as non-heat treatment precipitation-strengthened alloys is achieved primarily by solid solution strengthening along with work hardening rather than precipitation strengthening. Therefore, severe plastic deformation (SPD) techniques can be often used to produce ultrafine-grained structures to fabricate ultra-high strength aluminum alloys. However, this approach often achieves the strengthening of material at the cost of reduced ductility. This paper comprehensively summarizes the various approaches of ultrafine/nanocrystalline materials for enhancing their plasticity, elaborates on the creation of a bimodal microstructure within the alloy, and discusses the formation of a nanotwin microstructure within the alloy and the incorporation of dispersed nanoparticles. The mechanisms underlying both the strengthening and toughening during large plastic deformation in aluminum alloys are summarized, and the future research direction of high-performance ultrafine crystalline and nanocrystalline Al-Mg aluminum alloys is prospected.

## 1. Introduction

Aluminum alloys have been applied in various fields such as the automotive industry, high-speed rail transportation sector, aviation industry, construction sector, and defense/military industries, due to their low density, high strength-to-weight ratio, excellent corrosion resistance, good formability, high thermal and electrical conductivity, weldability, impact resistance, non-toxicity, non-magnetic properties as well as their recyclability and reusability features [1,2,3,4,5,6].

In these alloys, the 5xxx series aluminum alloys with low and medium Mg content (1~5 wt.%) are extensively employed in various structures, including pressure vessels, automobile body panels, ship structures, and exterior panels of military armored vehicles due to their exceptional corrosion resistance, weldability, machinability, and moderate strength properties. Magnesium (Mg) is the primary alloying element in 5xxx series aluminum alloys which belong to the non-heat-treatable deformed aluminum alloys [7,8]. It was found that the solubility of Mg in aluminum could reach 17.5 at.% at a eutectic temperature of 451 °C, compared to approximately 1.7% at room temperature [8]. In commonly utilized industrial-grade 5xxx series aluminum alloys, the Mg content generally does not exceed 5.5%. Due to a larger atomic radius difference between Mg and A1, Mg contributes significantly towards solid solution strengthening effects. Although the solid solution of Mg in aluminum is improved with the increase in temperature, these alloys exhibit poor aging responses and are typically unsuitable for strengthening through aging due to difficulties in the nucleation of precipitation phases resulting from limited core availability and the large precipitation phase sizes observed during the aging process. The Mg content in 5xxx series aluminum alloys typically exists in a supersaturated state, which remains stable at room temperature. Upon deformation and subsequent annealing, the solid solution can precipitate either a β(Mg_5_Al_8_) phase or a substable β1(Al_3_Mg_2_) phase. At lower temperatures, the β1 substable phase exhibits considerable stability and resists transformation into the equilibrium phase for an extended period of time. However, aging strengthening effects from β1- or β-phase precipitation along grain boundaries or shear zones are limited, thereby compromising the corrosion resistance. Consequently, the high strength in 5xxx series aluminum alloys is obtained primarily by solid solution strengthening and work hardening instead of heat treatment [9,10,11]. With the rapid advancement of modern industry, traditional 5xxx series aluminum alloys no longer fully satisfy practical production requirements and so developing enhanced efficiency Al-Mg alloys holds great significance.

Based on the issues and challenges faced by the researchers in different countries, various mechanisms of strengthening metals and alloys such as deformation strengthening, fine-grain strengthening, and solid solution strengthening are commonly employed to enhance material properties [12]. Extensive studies have demonstrated that relying solely on one or two mechanisms for improving material performance has limitations. Multiple mechanisms should be combined to obtain different strengthening effects [13,14,15,16]. Severe plastic deformation (SPD) has emerged as a promising technology in achieving these ultrafine-grained materials with exceptional properties.

Unlike conventional methods of plastic deformation, SPD applies a very high pressure, up to several GPa, resulting in grain refinement of bulk coarse crystalline materials down to submicron (0.1–1 μm) or even nanocrystalline (<100 nm) levels [14]. SPD is described by the Hall–Petch relationship (empirical equation) σ = σ_0_ + kd^−1/2^ to characterize the fine-grain strengthening. The relationship between the yield strength σ and the mean grain diameter d can be described by this mathematical expression, where d represents the average grain size and k and σ0 are material-specific constants. Here, σ denotes the material’s yield strength. Within a specific range of grain sizes (d within a certain range), the alloy can achieve stronger material properties due to the effect of finer grains. This method differs from traditional fine-grained strengthening methods that involve applying extensive amounts of severe plastic deformation to coarse crystalline materials until they reach submicron (0.1~1 μm) or even nanocrystalline scales (less than 100 nm) [14]. Depending on specific experimental processing paths and conditions, when the grain size is decreased to the submicron or nanoscales, the strengthening effect often surpasses pure fine-grain strengthening mechanisms and involves various synergistic mechanisms. As a result, SPD exhibits enhanced efficiency in terms of strengthening effects [17,18]. Gleiter et al. [19] first found nanocrystalline materials with grain sizes ranging from 1 to 100 nm. Subsequently, Valiev et al. [17] produced nanostructured materials with ultrafine/nanocrystalline grains by the SPD process. They found that nanostructured materials obtained through SPD exhibit distinct physical, chemical, and mechanical properties compared to alternative approaches for preparing nanocrystalline materials [19,20,21,22,23]. The strength of the alloy can be significantly enhanced through the SPD process; however, the deformed alloy exhibits low toughness. This is attributed to the inefficient dislocation storage capacity of the ultrafine crystalline or nanocrystalline structure in high-strain alloys, resulting in a reduced work hardening rate. Consequently, this limitation greatly hampers their practical applicability. To address this drawback, various approaches have been proposed in recent years to enhance the work-hardening rate of these alloys. The aim of this review is to elucidate the merits and limitations of SPD techniques applied to Al-Mg alloys, along with proposed remedies for its shortcomings, thereby providing novel insights for the future development of ultrafine crystalline and nanocrystalline Al-Mg alloys with high strength and toughness.

## 2. Severe Plastic Deformation (SPD) of Al-Mg Alloys

Severe Plastic Deformation (SPD) has emerged as a promising technique for achieving ultrafine-grained and nanostructured materials with excellent properties [24]. This process involves (i) the repetitive plastic shear deformation of the material and (ii) the transformation of coarse grains into nanometer-scale grains. Nanocrystalline metallic materials produced by large plastic deformation technique exhibit a high strength due to layer dislocations, nanotwins, and nonequilibrium grain boundaries [25,26,27]. Moreover, these materials with a small grain size and high defect density have many excellent properties that are incomparable with traditional coarse crystalline metallic materials [27,28,29,30,31].

In addition, single-phase Al-Mg alloys processed by SPD are usually extremely susceptible to the coarsening of fine crystals at elevated temperatures with their poor thermal stability [32,33], leading to increased difficulty in controlling their superplastic organization [34,35,36,37,38,39]. Therefore, the reorientation of alloy grain boundaries via SPD processing has emerged as a promising strategy for enhancing the thermal stability of the grain structure [40,41,42].

To date, more than 30 SPD techniques have been proposed. Among the various SPD techniques, High Pressure and Torsion (HPT) [43,44,45,46,47], Equal-Channel Angular Pressing (ECAP) [48,49,50,51,52,53], and Accumulative Roll Bonding (ARB) [54,55,56,57,58,59,60] are most prominent.

### 2.1. High Pressure and Torsion

**Figure 1:**
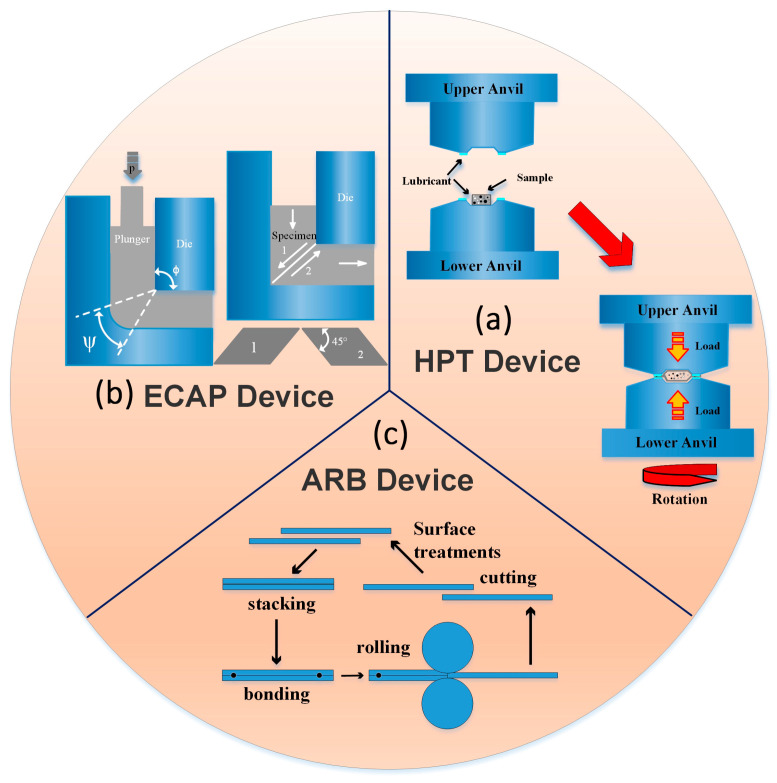
(**a**) HPT device, (**b**) ECAP device, (**c**) ARB device.

High pressure and torsion (HPT), first introduced by Straumal et al. [61,62], as shown in Figure 1a [62], is an SPD technology where a sample is subjected to torsional shear straining.

The HPT device consists of three main parts: the pressure system, the upper die, and the lower die [63]. The sample is placed in the groove between the upper die and lower die during operation. Then, the sample is extruded and deformed in the axial direction and fixed to the die under the applied pressure of the GPa level to the upper die. By twisting the lower die [64], the sample located in the middle of the upper and lower dice is subjected to an axial compressive stress, a horizontal friction force (where the sample is in contact with the upper and lower dice), and a shear stress at the same time. The plastic deformation occurs when a material is subjected to the three stresses that exceed its yield strength, and will result in the appearance of a significant amount of internal displacements within the material and the formation of a large number of ultrafine crystals or even nanocrystals in the material, leading to a substantial enhancement in the strength of materials. Therefore, the HPT technique is the effective method for SPD; however, the processed samples are limited to the size of a coin and the shape of a disk, and the microstructure of the processed material is also not homogeneous [65].

**Figure 2:**
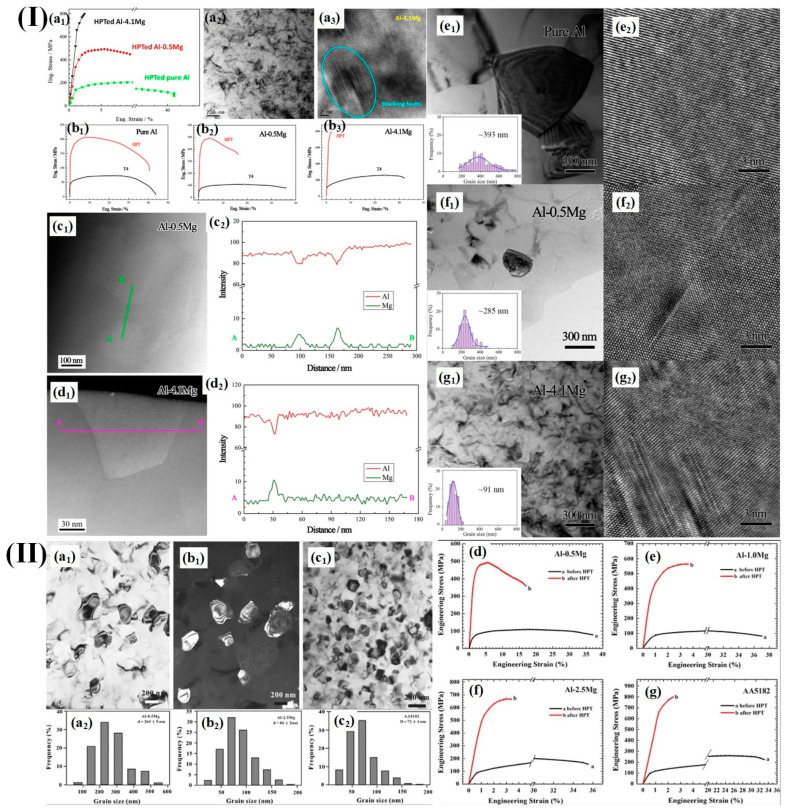
(**I**) (**a1**–**a3**) Engineering stress–strain curves and corresponding transmission electron microscopy images of HPTed samples; (**b1**–**b3**) engineering stress–strain curves of blank and HPTed samples; (**c1**–**d2**) HAADF-STEM images and linear scanning EDX analysis on grain boundaries of blank and HPTed samples; (**e1**–**g2**) transmission electron microscopy images of HPTed samples [66]; (**II**) (**a1**–**c1**) transmission electron microscopy images of HPTed samples; (**a2**–**c2**) histograms depicting the distribution of grain sizes in HPTed samples; (**d**–**g**) engineering stress–strain curves of blank and HPTed samples [67].

In recent years, many studies have obtained ultrafine crystalline and nanocrystalline materials using the HPT technique. For example, Liu et al. [66] demonstrated the remarkable strengthening effect of A1-Mg alloys prepared through HPT. In particular, as depicted in Figure 2I, the Al-4.1 wt.% Mg alloy exhibited an exceptionally high tensile strength of approximately 800 MPa.

This enhancement can be attributed to the formation of ultra-fine grains (with an average size of around 91 nm) induced by deformation, as well as the presence of high-density laminar dislocations and the segregation of Mg. It is worth noting that the mechanical performance of HPT alloys not only depends on severe plastic deformation, but also is influenced by the magnesium content [67]. Both hardness measurements and tensile tests revealed that the mechanical properties of deformed Al-Mg samples are significantly higher than that of undeformed counterparts. Furthermore, there was a notable increase in both hardness and strength for HPT-treated samples with higher levels of magnesium content (Figure 2II). Tan et al. [68] performed high-pressure torsion experiments on Al-13.4wt.%Mg at a pressure of 6 Gpa. They found that the Vickers hardness of torsion samples under the high-pressure reached 270 HV, which was increased by 150 HV compared to that of the sample without high pressure and torsion.

**Figure 3:**
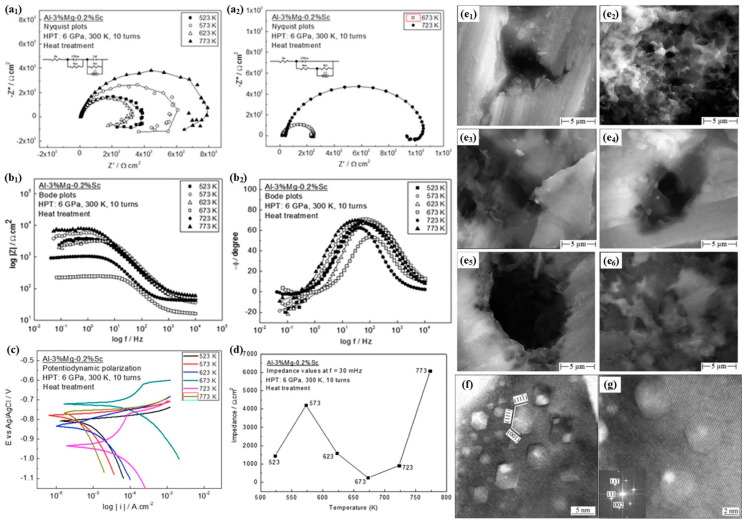
(**a1**–**b2**) Nyquist diagrams of the alloy following heat treatment in a 3.5% (wt.%) NaCl solution after stabilizing at the open circuit potential for 1 h; (**c**) potentiodynamic polarization curves were obtained for the alloy immersed in a 3.5% (wt.%) NaCl solution; (**d**) Al–Mg–Sc alloy subjected to HPT processing and subsequent annealing exhibited impedance values at a frequency of 30 mHz; (**e1**–**e6**) SEM was used to examine the surfaces of the alloy after undergoing HPT processing and subsequent annealing [69]; (**f**,**g**) nanostructures with a high density and hexagonal or rhombic shapes in aluminum–magnesium alloys subjected to HPT [70].

Lage et al. [69] observed that the grain size distribution of Al-3%Mg-0.2%Sc alloys treated with HPT was impacted by annealing temperature and also investigated the corrosion resistance (Figure 3).

It was found that the annealed samples at 673 K were composed of larger and more heterogeneous grains, leading to lower impedance values. Conversely, Al alloys annealed at 773 and 573 K demonstrated superior oxidation resistance and charge transfer resistance among the treated alloys. Moreover, polarization analysis revealed both Al-Mg-Sc alloys annealed at 573 and 773 K had minimal corrosion current density when they were exposed to brine medium. Notably, the Al-Mg-Sc alloy annealed at 773 K can have exceptional corrosion resistance among all tested conditions. High-density hexagonal and rhombic-shaped nanostructures are commonly observed in aluminum–magnesium alloys subjected to high-pressure torsion [70] (Figure 3f,g).

### 2.2. Equal-Channel Angular Pressing (ECAP)

The ECAP technique was first introduced by Segal et al. Subsequently, many researchers have further explored this technique for different materials. Valiev et al. [71,72,73,74,75,76,77,78,79] and Segal et al. [80,81,82,83] have made substantial advancements in this research domain. For example, Valiev et al. effectively attained ultrafine equiaxial crystalline structures of various materials including pure metals, alloys, and intermetallic compounds by the equiaxial angle extrusion technique [72,73,74,75,76,77,78,79,80]. The principle of ECAP is illustrated schematically in Figure 1b [84], which is composed of a hydraulic press that can provide a stable pressure and a die. The extrusion die is a pipe with a uniform internal diameter, which is bent in the middle to a 90° shape. During the experiment, a constant compressive stress is exerted on a cylindrical sample by a hydraulic press, where the diameter is slightly less than that of the pipe that is inserted into the pipe. In the bending flexural test, the specimen is bent to 90° through the middle under shear stress, but the shape of the specimen does not change significantly after passing through the molded pipe. Therefore, the specimens can be extruded by ECAP up to multiple passes to acquire a desirable ultrafine-grained microstructure. The multi-pass extrusion process can also have a rotation of 90° or 180° to achieve different extrusion effects.

**Figure 4:**
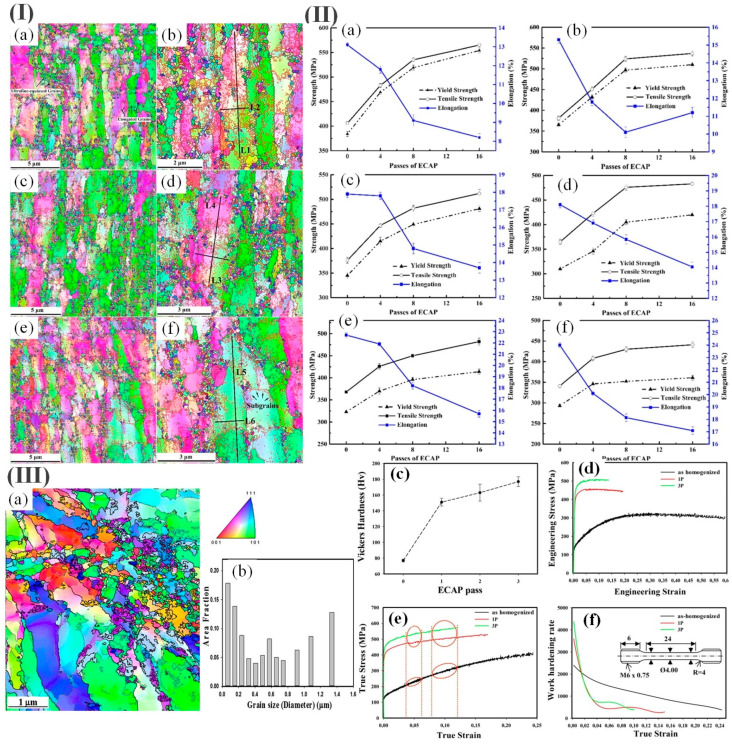
(**I**) EBSD maps were obtained for the Al-Mg-Sc-Zr alloy after undergoing 16 ECAP passes + CR, followed by annealing at various temperatures for a duration of 1 h: (**a**) without annealing and (**b**) enlargement of (**a**); (**c**) 200 °C and (**d**) enlargement of (**c**); (**e**) 240 °C and (**f**) enlargement of (**e**); (**II**) the evaluation of the mechanical characteristics of Al-Mg alloy and Al-Mg-Sc-Zr alloy after undergoing various ECAP + CR passes and annealing at different temperatures for a duration of 1 h: (**a**) Al-Mg, without annealing; (**b**) Al-Mg-Sc-Zr, without annealing; (**c**) Al-Mg, at 240 °C; (**d**) Al-Mg-Sc-Zr, at 240 °C; (**e**) Al-Mg, at 280 °C; (**f**) Al-Mg-Sc-Zr, at 280 °C [86]; (**III**) (**a**) ASTAR-TEM orientation images of the 3P ECAP material; (**b**) the distribution of grain sizes is analyzed in terms of area fractions; (**c**) Vicker’s hardness measurements are conducted on an Al_7_Mg alloy as the number of ECAP passes increases; (**d**) the engineering and true tensile stress–strain (**e**) curves for the Al_7_Mg alloy are plotted against increasing ECAP passes; (**f**) the work-hardening rate is examined as a function of true strain [87].

Haghayeghi et al. [85] investigated the impact of equal-channel angular pressing on the mechanical characteristics of Al-6Mg alloy and they found that this process can successfully enhance the tensile strength up to 550 MPa. According to Tang et al. [86], Al-Mg-Sc-Zr aluminum alloys were manufactured by 16 passes of equal-channel angular pressing (ECAP) treatment followed by annealing at various temperatures. The results (Figure 4II) indicated that the aluminum alloys exhibited a yield strength of approximately 481 MPa, an ultimate tensile strength of around 512 MPa, and an elongation of about 13.7% after the optimal annealing process (240 °C × 1 h). However, there was no significant growth of ultrafine equiaxed grains surrounding the coarse grains even after annealing at different temperatures (200 °C and 240 °C) (Figure 4I).

Zha et al. [87,88] found that more than three extrusion passes of ECAP of Al-7Mg alloys at room temperature resulted in a large number of cracks leading to material failure [87]. In contrast, Zha et al. [88] reported that the hard-to-transform Al-7Mg alloy was processed in six passes by ECAP, combined with intermediate annealing. The Electron Back Scatter Diffraction (EBSD) results revealed a relatively homogeneous structural arrangement in Al-1Mg alloys, whereas a mixed crystalline organization with the coexistence of coarse crystals (tens of micrometers) and fine crystals (<500 nm) occurred in Al-5Mg and Al-7Mg alloys with high Mg content. Moreover, the size of medium-axial fine crystals in Al-7Mg alloy is obviously finer than those in Al-5Mg alloy. The tensile deformation behavior of Al-Mg alloys after ECAP demonstrates that the strength and plasticity of Al-7Mg alloys are higher than those of Al-1Mg and Al-5Mg alloys. Moreover, the deformed Al-7Mg alloys exhibit exceptional ductility (14.5%) along with ultra-high strength (600 MPa) [88]. The remarkable strengthening by post-ECAP-treated Al-7wt.% Mg alloy can be attributed to a combination of factors including increased dislocation density, the formation of ultrafine grains, and enhanced magnesium solute content. Figure 4III shows that the application of room temperature equal-channel angular pressing (ECAP) processing can lead to the development of Al-7wt.%Mg alloys with exceptional mechanical properties. These alloys exhibit a tensile strength of 509 MPa and a high homogeneous elongation of 14% after undergoing three passes.

**Figure 5:**
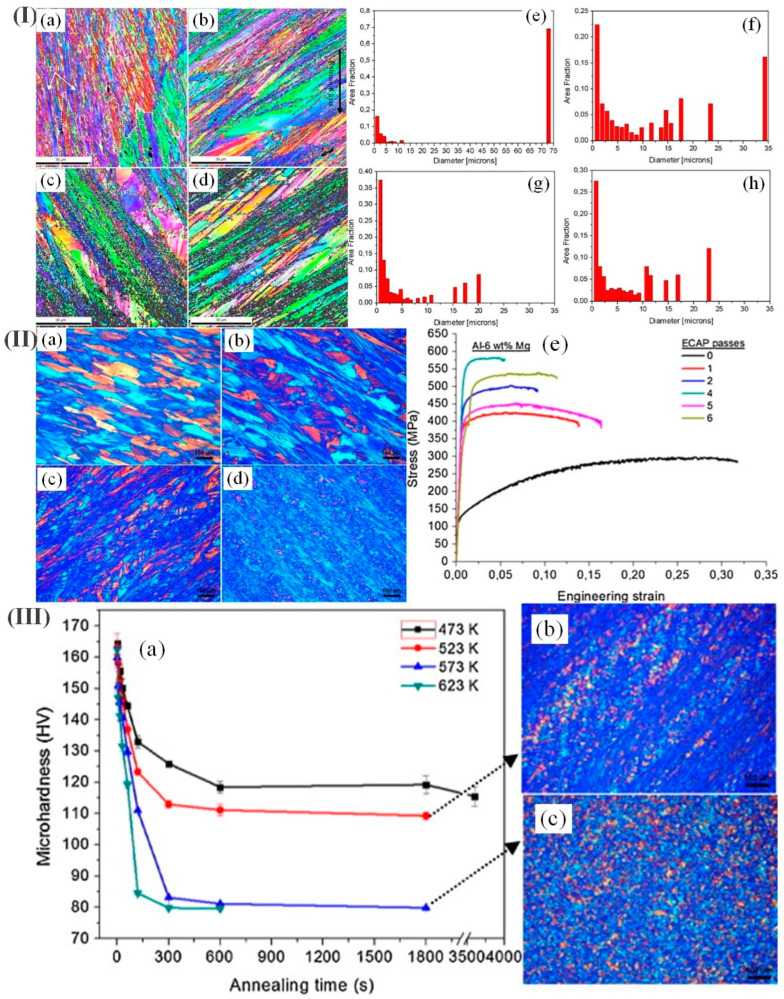
(**I**) (**a**–**d**) Characterization using SEM-EBSD technique was conducted on Al–xMg alloys subjected to 3 ECAP passes at ambient temperature; (**e**–**h**) grain size distributions were analyzed for the same set of Al–xMg alloys after undergoing 3 ECAP passes; (**II**) (**a**–**d**) POM microstructures were observed in an annealed ECAP process applied to an Al-6wt%Mg alloy under ambient conditions; (**e**) an investigation of the mechanical behavior of an Al-6 wt% Mg alloy subjected to annealed ECAP processing; (**III**) (**a**) variations in the hardness of an Al-6 wt% Mg alloy were observed after undergoing 6 rounds of annealed ECAP, with respect to different durations of annealing. Additionally, microstructural changes were examined following a 30 min annealing process at (**b**) 523 K and (**c**) 573 K [89].

Chen et al. [89] indicated that annealing was performed at 250 °C for 5 min and at 350 °C for 10 min and that between 2 and 4–5 passes of angular pressing may avoid the formation of the cracking of Al-6 wt.%Mg alloy during the equal-channel angular pressing process. The mixed crystal structure containing coarse crystal grains and exquisite particles in the alloy is obtained. As depicted in Figure 5I, a negative correlation exists between the Mg content and the size of the fine grains.

The ECAP treatment at different ambient temperatures resulted in grain refinement for all Al-xMg alloys through an increased occurrence and interaction of shear bands. Figure 5II(e) shows that the yield strength increased from 127.4 MPa to 562.2 MPa, while the elongation experienced a notable reduction from 31.7% to merely 5.5% following four ECAP extrusions. The changes in the softening mechanism were observed within a temperature range of 523–573 K for Al-6wt%Mg alloys treated with six rounds of annealing-based ECAP (Figure 5III).

The evolution of shear planes and directions induced by different routes in the material defines grain refinement during the process of equirectangular extrusion [90,91,92,93]. Moreover, elevating the extrusion temperature can lead to a noticeable increase in grain size, consequently reducing the yield stress of the material [94,95,96]. Conversely, cutting speed has no obvious influence on grain size but does affect its distribution to some extent [97,98]. Although the ECAP technology can improve the mechanical properties by subjecting the material to SPD, it is limited by the complexity of the pressing process and the size of the pressed material, which prevents mass production.

### 2.3. Accumulative Roll Bonding

**Figure 6:**
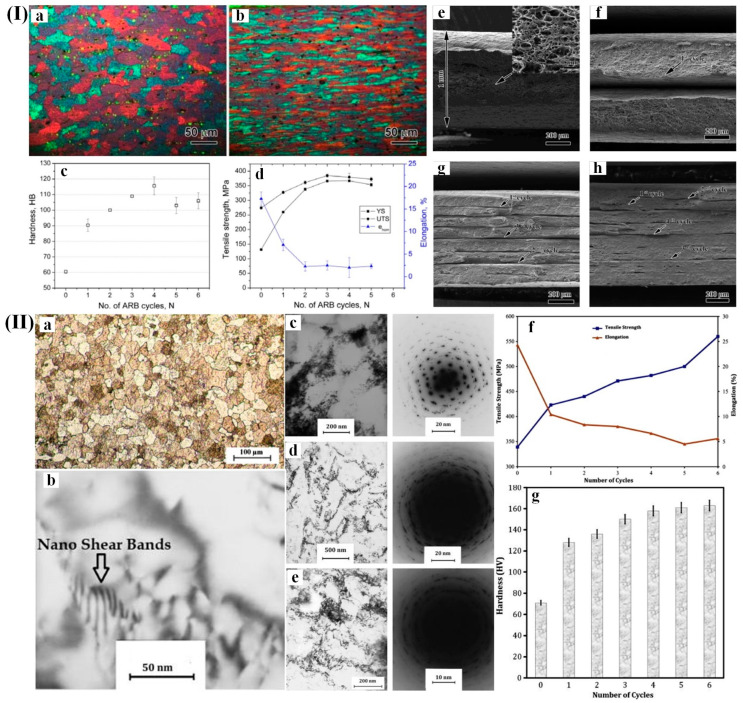
(**I**) (**a**,**b**) Micrographs illustrating the examination of Al-Mg alloy sheets (**a**) prior to undergoing the ARB process, and (**b**) after undergoing 6 cycles of ARB; (**c**) variations in the hardness (HB); (**d**) tensile properties (El, YS, UTS) are observed as the number of ARB cycles increases; (**e**–**h**) fracture surfaces of Al-Mg alloy sheets during tensile testing conditions; (**II**) (**a**) the characterization of the fully annealed AA5083 strip prior to undergoing the accumulative roll bonding procedure; (**b**) repetitive deformation cycles induce the formation of nano shear bands in the AA5083 strip; (**c**–**e**) TEM micrographs and corresponding SAD patterns of the AA5083 strip following: (**c**) the second, (**d**) the fourth, and (**e**) the sixth cycles; (**f**) variations in the strip’s tensile strength and elongation for different cycles of AA5083; (**g**) hardness variation in the AA5083 strips across different cycles [103].

The accumulative roll bonding (ARB) technique (invented by Saito [99]) has attracted much attention in recent decades due to its high potential in introducing fine-grain structure into various metals during multi-step rolling. The schematic representation of the ARB device is shown in Figure 1c [100] and its process is observed in Ref. [101]. A rectangular piece, with uniform thickness, is first taken from the sheet metal, and then two parts of equal size cut from the middle and surfaces of two samples are polished. The two pieces are stacked and fixed at both ends at the same time to avoid relative sliding during the rolling process, and then they are rolled to 50% of the pressed down amount by the rolling mill at one time. The materials only undergo changes in thickness reduction and elongation along rolling direction because the rolling is subjected to forces in only the rolling direction and the direction perpendicular to the surface. The length of material after rolling is equal to the initial rectangular length because the material can be repeatedly rolled to the desired amount of deformation. Alil et al. [102] reported that the initial 5083Al alloy (Al-4.16 wt.% Mg) sheet obtained by hot rolling underwent a cold-rolling process, resulting in specimens with a reduced average thickness of 1.65 mm. Subsequently, the aluminum alloy sheet was subjected to a cold rolling process, resulting in a thickness of 1 mm; subsequently it underwent annealing at a temperature of 320 °C for a duration of 3 h. The alloy sheets of a l mm thickness were fabricated by cumulative cold rolling with intermediate annealing treatment at 320 °C for 5 min; ultimately, six cumulative rolling passes were performed. The average grain size reduced from 23.13 ± 0.29 μm to less than 500 nm after six passes of cumulative rolling. Figure 6I(c) shows that the hardness of the alloy increased from 60 HB to 115 HB and the initial yield strength increased from 130 MPa to 370 MPa after four passes of cumulative rolling, respectively. Similarly, the tensile strength also demonstrated an enhancement, increasing from 275 MPa to 385 MPa (Figure 6I(d)).

Mohammad et al. [103] reported that the grain size of AA5083 strips decreased from 25 μm to 80 nm by ARB technique. The SAD pattern exhibited a more toroidal shape with an increase in the number of cycles, indicating an augmentation in high-angle grain boundaries (Figure 6II(b)). Furthermore, nano shear bands appear within the microstructural framework after four cycles, as we can see in Figure 6II(d). In terms of mechanical properties, there was a significant enhancement in tensile strength for AA5083 strips with an increasing number of ARB cycles (Figure 6II(f)). The elongation values experienced a decrease throughout most cycles, except for the final one. Notably, there was a substantial increase in hardness value from 71 to 163 HV for AA5083 strip samples after completing six cycles, representing an impressive boost of approximately 130% (Figure 6II(g)).

The assessment of structural material quality heavily relies on strength and ductility. However, simultaneously enhancing the strength and ductility of metals and alloys has been a tremendous challenge. In the past few decades, tremendous efforts have been dedicated to developing structural materials due to their exceptional strength alongside remarkable plasticity.

From the perspective of dislocation motion, the high strength of alloys arises from hindering dislocation motion [104,105,106], whereas the improvement of their plasticity is associated with the spatial distribution, proliferation, and propagation of dislocations [106,107,108,109,110]. Consequently, it is necessary to overcome the strength–plasticity trade-off, implying that these two pivotal performance indicators are perpetually at odds with each other [111]. Compared with coarse crystalline alloys, nano-/ultrafine crystalline alloys generally demonstrate superior strength characteristics; however, they often exhibit limited plasticity and lack work-hardening ability due to their inadequate capacity for storing dislocations efficiently. This shortcoming significantly limits their potential for practical applications [112]. For ultrafine-grained Al-Mg alloys fabricated using SPD technology, the abundance of internal dislocations within individual grains is proved to be unfavorable to effective dislocation accumulation during tensile processes. The ultrafine grains exert an image force on these internal dislocations that attracts and traps them at grain boundaries. As a result, the efficient accumulation of dislocations within individual grains is impeded, leading to a reduced rate of work hardening in ultrafine-grained Al-Mg alloys even at low strains—ultimately resulting in premature necking and fracture [113,114,115].

**Figure 7:**
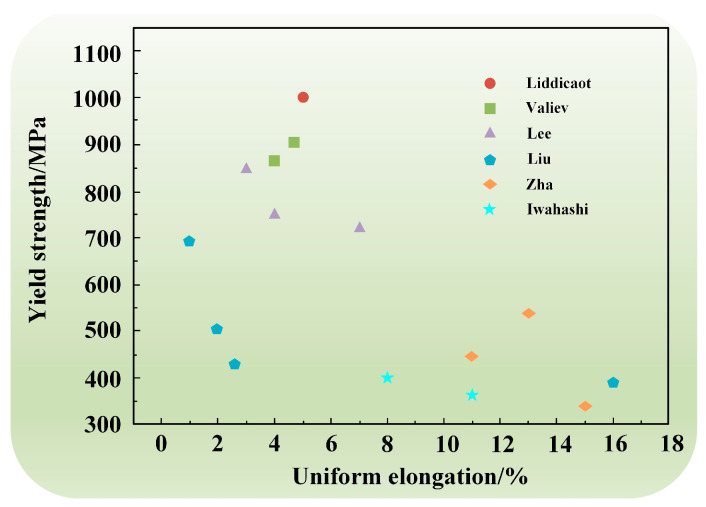
Strength–ductility of Al alloys processed by SPD.

In recent years, a large number of studies have shown that the work-hardening rate of alloys can be enhanced by various approaches. For example, introducing a bimodal microstructure can provide plasticity while nanograins provide strength [31]. Moreover, introducing a nanotwin microstructure inside the material could accommodate numerous dislocations and improve the work-hardening rate [116]. The incorporation of dispersed nanoparticles could effectively prevent dislocation motion to improve the work-hardening rate [117]. For comparison, the strong toughness of several typical SPD aluminum alloys is summarized in Figure 7 [67,87,118,119,120,121].

## 3. The Influence of a Bimodal Microstructure on the Properties of Al-Mg Alloys and Its Formation Mechanism

In general, SPD-induced ultrafine-grained (UFG) and nano-sized grains exhibit diminished work-hardening capacity during plastic deformation owing to their reduced dislocation storage capability and enhanced dynamic recovery of non-equilibrium grain boundaries [57].

Nanocrystalline materials with a grain size less than 100 nm exhibit exceptional mechanical properties such as high strength and wear resistance due to their unique structure. However, the reduction in grain size generally leads to a decrease in the ductility of these materials, thereby limiting their potential engineering applications [122,123]. Over the past decades, valuable efforts have been devoted to enhance the ductility of nanocrystalline materials. Introducing a bimodal grain-size distribution has been demonstrated as an efficient strategy for fabricating high-strength and ductile metallic materials by embedding micron-sized coarse grains within the nanocrystalline matrix. The grain size follows a statistical bimodal distribution, characterized by a probability density function exhibiting distinct peaks [124,125]. The coarse crystal exhibits a high dislocation accommodation capacity, an extended range of dislocation slip, and a reduced number of slip barriers, thereby effectively enhancing the work-hardening ability and improving the material’s plasticity. Additionally, the presence of fine crystal organization within the mixed crystal structure can significantly enhance the strength of alloys [126,127,128,129,130,131,132,133,134]. Consequently, regulating the alloy to form a mixed crystal structure can simultaneously enhance both material strength and plasticity. Numerous researchers have qualitatively confirmed that a bimodal grain size structure can effectively enhance the ductility of nanocrystalline materials.

**Figure 8:**
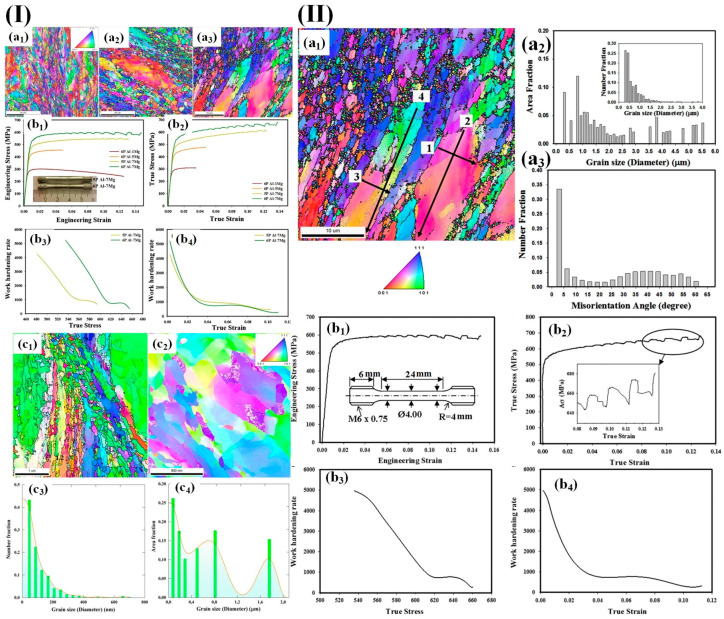
(**I**) FESEM-OIM mappings were conducted on samples of (**a1**) 6P Al-1 Mg, (**a2**) 6P Al-5 Mg, and (**a3**) 6P Al-7 Mg in the ND-ED region; (**b1**) stress–strain curves for ECAP Al-xMg samples in engineering conditions and (**b2**) stress–strain curves in true conditions, along with the work-hardening rate as a function of (**b3**) true stress and (**b4**) true strain for the Al-7 Mg samples subjected to 5P and 6P ECAP; (**c1**,**c2**) ASTAR-TEM orientation images at lower magnification and (**c3**,**c4**) corresponding grain size distributions of the 6P Al-7 Mg sample [88]; (**II**) (**a1**) a representative orientation map in the ND-ED plane of the Al-7Mg alloy under investigation is presented. (**a2**) The distribution of grain sizes and (**a3**) the chart illustrating the distribution of misorientation angles are derived from the aforementioned orientation map; (**b1**) the stress–strain curve for the current Al-7Mg alloy, (**b2**) the true stress–strain curve, and the work-hardening rate curve as a function of (**b3**) applied stress and (**b4**) deformation [135].

Zha et al. [87,88,135] prepared the Al-7Mg alloys by room temperature equal-channel angular pressing (ECAP). They found that the microstructure of the alloys consisted of a significant number of ultrafine crystals with an average size of approximately 500 nm and some coarse crystals. These ultrafine crystals accounted for around 70% of the overall microstructure, exhibited high strength (~570–600 MPa), and excellent uniform elongation (~11–14%) (Figure 8).

Moreover, an increased Mg content can effectively hinder dislocation glide during tensile processes, thereby enhancing the strength of the alloy. Simultaneously, a higher Mg content facilitates the formation of a mixed crystal structure and the fine crystalline component contributes to the improvement in strength. The coarse crystalline portion within this mixed crystal structure possesses ample capacity to accommodate numerous dislocations, consequently enhancing both the work-hardening ability and plasticity of the alloy. Lavernia et al. fabricated Al-7.5Mg and AA5083 alloys with a hybrid crystalline structure through a low temperature ball milling and a hot isostatic pressing process, demonstrating their exceptional combination of high strength and plasticity [136,137]. Lavernia et al. found that coarse crystals in the organization of the mixed crystal structure can inhibit crack initiation and growth, thus delaying fracture. Meanwhile, the coarse crystal has a large dislocation accommodation capacity, a longer dislocation slip range, and fewer slip barriers. The incorporation of this technique effectively enhances the capacity to withstand work hardening and augments its pliability. The fine crystal organization in the mixed crystal structure organization can effectively enhance the durability of the alloy.

**Figure 9:**
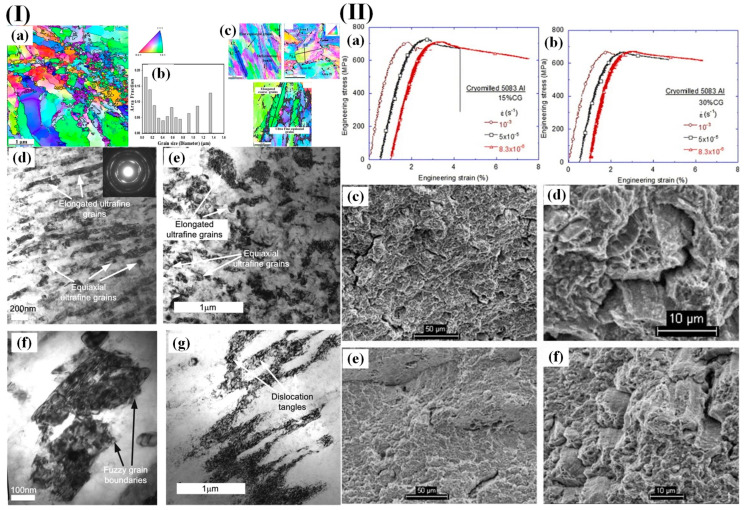
(**I**) (**a**–**c**) Orientation images of the 3P ECAP material obtained using ASTAR-TEM; (**b**) the distribution of grain sizes represented as area fractions; (**d**–**g**) TEM images depicting the material processed through 3P ECAP [87]; (**II**) the tensile performance of cryomilled 5083 aluminum alloy was evaluated under different strain rates, with (**a**) a composition containing 15% coarse grains and (**b**) a composition containing 30% coarse grains; fracture analysis was conducted on the cryomilled 5083 Al alloy with an initial grain size of 30% larger grains under two different strain rates: (**c**,**d**) 10^−3^/s and (**e**,**f**) 8.3 × 10^−6^/s [128].

Zha et al. [87] analyzed the ECAP mechanism in high solid solution Al-7Mg alloys at room temperature for deformation. It was observed that the coarse equiaxial crystals in the homogeneous state were disrupted after a single pass of deformation, resulting in elongated grains at an angle of 60° relative to the extrusion direction and at grain boundaries. Refined deformation bands and a small number of fine equiaxial grains were formed at both regular grain boundaries and triangular grain boundaries. After two passes through the ECAP process, numerous deformation bands formed within the coarse grains while there was also an increase in isometric grain count. Further increasing passes through the ECAP process resulted in further refinement of the grains, with chain-like formations of fine crystals surrounding larger-angle grain boundaries from remaining coarser crystals, ultimately yielding a typical mixed crystal structure organization consisting of micrometer-sized coarse crystals alongside ultrafine ones measuring less than ~500 nm (Figure 9I).

With increasing ECAP passage, the coarse grains will gradually transform into fine grains that are bounded by high-angle grain boundaries (HAGB). The ultrafine grains and/or subgrains exhibit fuzzy grain boundaries and dislocation tangles, indicating a significant local strain and a high density of dislocations. Moreover, Figure 9I(g) illustrates the presence of numerous dislocation tangles in the subgrain structure of coarse grains, further confirming their high dislocation density. Han et al. [128] investigated the variation in tensile ductility of a bimodal 5083 aluminum alloy subjected to low-temperature milling at different strain rates (Figure 9II(a,b)). They revealed a linear increase in uniform strain, while the ductility exhibited a significant enhancement with decreasing strain rate. Furthermore, distinct disparities were observed in the fracture surface morphology under varying strain rates, indicating interfacial delamination and pit formation within regions characterized by coarser grains at higher strain rates. In particular, necking specifically occurred within these coarse-grained areas, suggesting localized ductile damage within them. Moreover, Figure 9II(d) illustrates that delamination occurs at the interface between coarse-grained and nanostructured regions.

**Figure 10:**
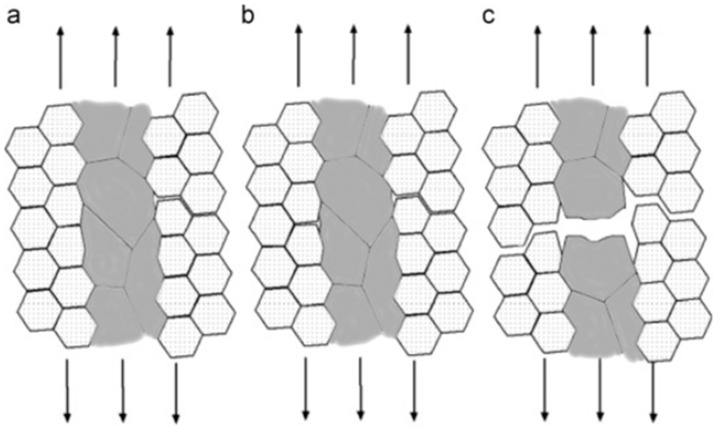
(**a**–**c**) The propagation of cracks within the microstructure exhibiting a bimodal distribution [137].

The addition of fine grains in mixed crystals effectively enhances the strength of alloys, while the presence of coarse grains (~1 μm) inhibits crack propagation and improves plasticity (Figure 10).

Cracks initially initiate within the fine-grain region and then propagate along grain boundaries. However, when these microscopic cracks extend to the coarse-grain boundaries, their further growth will be impeded by the coarse grain. With an increasing number of cracks in the fine-grain region that propagate into the coarse grain, the accumulation of cracks leads to hole formation within the coarse-grain region, ultimately resulting in alloy cracking. Therefore, organizing a mixed crystal structure significantly enhances alloy plasticity.

## 4. The Influence of the Formation of Nanotwin Microstructure within Al-Mg Alloys on Their Properties and Its Mechanism

Subgranular boundaries, grain boundaries, and phase interfaces, with their diversity in both structure and structural transitions, play a crucial role in determining the properties of polycrystalline materials. Among them, twin crystals are a special type of interface that significantly influence material properties. Twins are two crystals or two parts of a crystal that exhibit a mirror-symmetric dislocation relationship along a shared crystal plane, with a specific orientation relationship. It has been experimentally demonstrated that twins effectively impede dislocation motion. In general, twins in metals can be classified into deformation twins, growth twins, and annealed twins based on their formation methods [138]. Twinned metals commonly exhibit excellent properties such as high strength, excellent electrical conductivity, superior toughness, and enhanced thermal stability. Therefore, the generation of stable twinning structures in metals is considered an effective approach to enhance the mechanical properties of materials.

The formation of twins in metals is closely associated with the energy of layer dislocations. Previously, it was believed that metals with low layer dislocation energies such as silver [139], copper [140,141], and nickel [142] are more prone to twin formation. For example, Lu et al. [143] have demonstrated the facile generation of high-density growth twins in copper by electrodeposition and sputtering experiments. However, the formation of twin crystals in aluminum poses a challenge due to its high layer dislocation energy [144,145]. Molecular dynamics (MD) simulations have revealed that laminar dislocations/twins can only be formed in aluminum grains with sizes smaller than 35 nm [146]. Furthermore, it has been observed that laminar dislocations/twins can also occur in aluminum materials under conditions of significant deformation.

**Figure 11:**
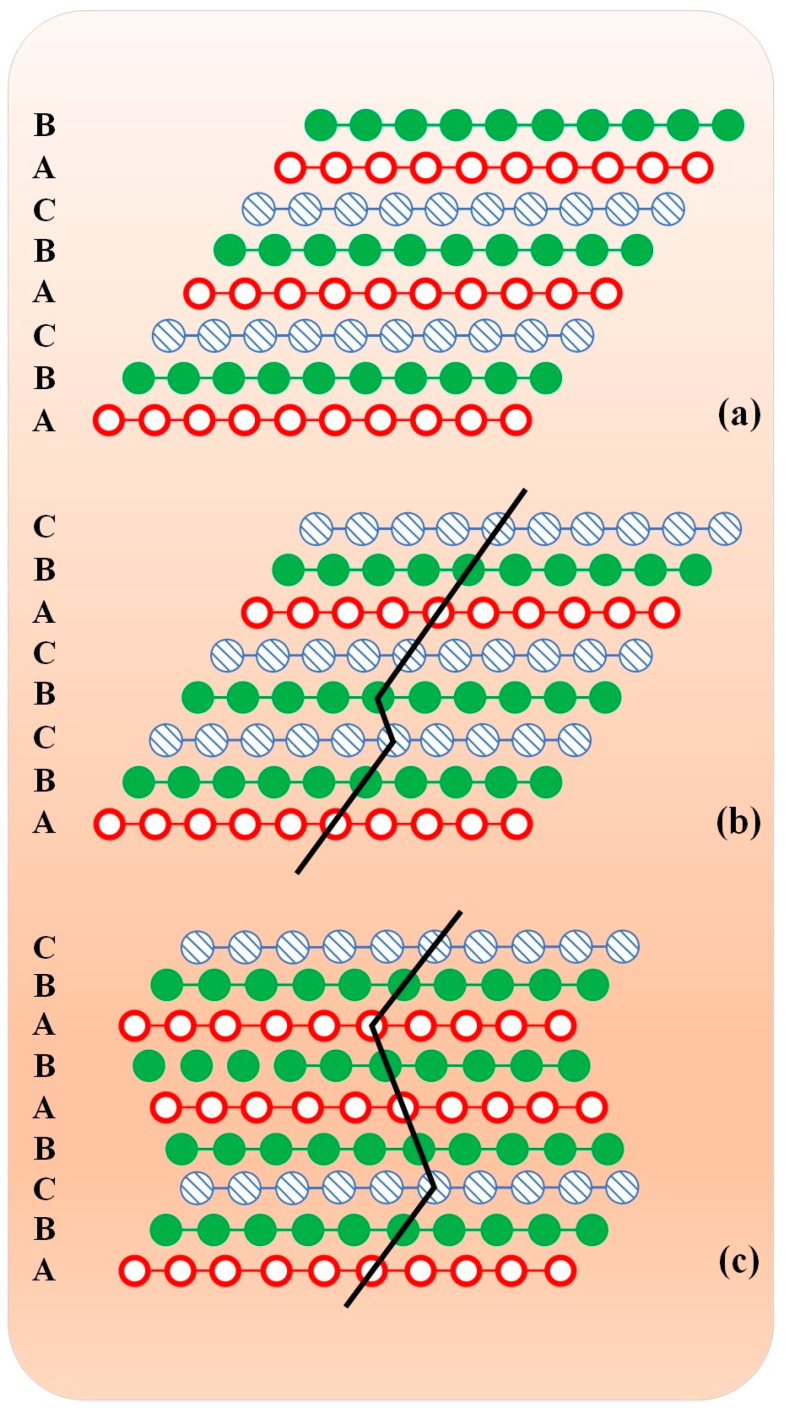
(**a**–**c**) Schematic illustration of twin formation through dynamic superposition of layer faults in FCC metals.

The schematic diagram in Figure 11 illustrates the formation of twinned crystals in face-centered cubic (FCC) metals through the dynamic stacking of layer faults. In Figure 11a, the lattice projection along the (110) pin direction has no crystal defects in the FCC metal and the stacking order follows the normal ABCBCABCABC sequence.

Figure 11b demonstrates that a full dislocation inside the grain dissociates into Shockley partial dislocations to form a layer dislocation, resulting in a change in the stacking order from ABCBCABCABC to ABCBCABC. Continuing this process as depicted in Figure 11c, a nanotwin crystal is formed by stacking four nanotwin crystals generated from four layer dislocations, with a modified stacking order of ABCBABABC.

**Figure 12:**
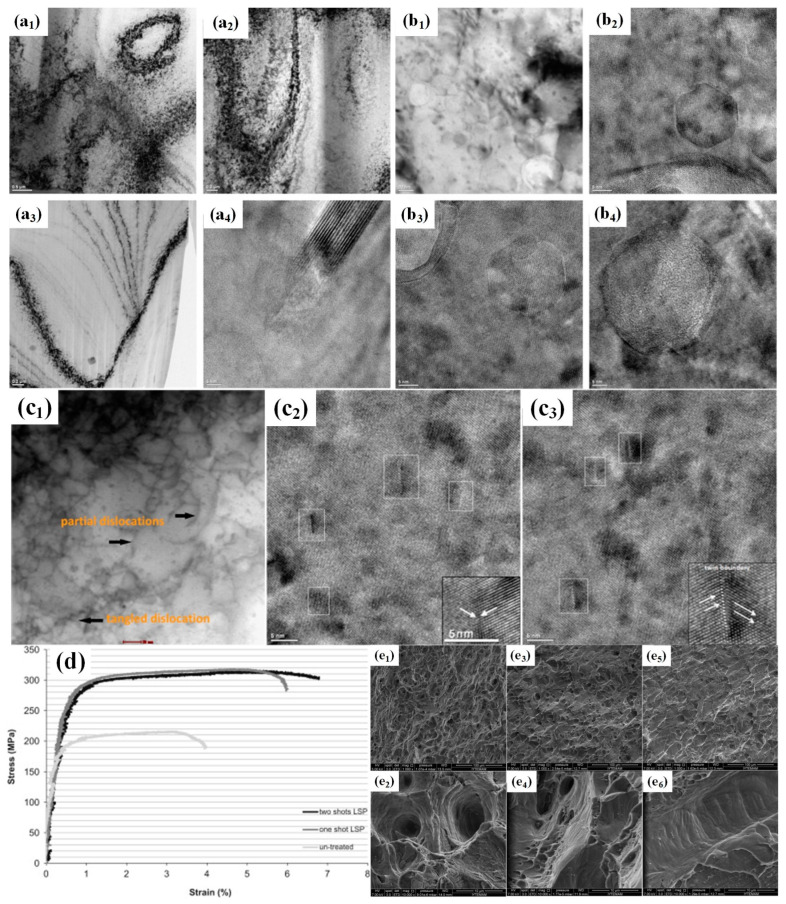
(**a1**–**a4**) display TEM images of a sample treated with one-shot LSP; TEM images acquired from (**b1**) a sample treated with two-shot LSP, demonstrating nanocrystalline structures (**b3**) and atomic-level resolution (**b2**–**b4**); TEM images captured from a sample treated with LSP, illustrating (**c1**) interlaced dislocations and partial dislocations, and (**c2**,**c3**) numerous deformation twins; (**d**) displays the stress–strain curves of untreated and LSP-treated alloys; (**e1**–**e6**) picture of the rupture [148].

Laser Shock Peening (LSP) is a highly effective mechanical surface modification technique. For example, Irizalp et al. [147] significantly enhanced the strength and plasticity of 6061-T6 aluminum alloy by laser shock treatment. Experimental results demonstrated that the tensile strength of 6061-T6 aluminum alloy increased from 220 MPa to 320 MPa after laser impact treatment, while the strain to failure rose from 4% to 7% (Figure 12d).

The enhancement in both strength and plasticity may be attributed primarily to the refinement of aluminum grain size and presence of surface defects such as layer error and twinning. Upon reaching a sufficient number of dislocations in the material, these dislocations become entangled and aggregate into large clusters (Figure 12(a1,a2)).

Furthermore, interactions among dynamic dislocations result in the formation of cells (Figure 12(a3)). Additionally, stacking faults (SFs) were observed in the TEM images (Figure 12(a4)). Figure 12(b1–b4) give not only the formation and accumulation of twins but also the generation of nanocrystals. Figure 12(b2–b4) exhibits atomic-resolution views of the nanocrystal structure. The microstructure exhibits a high degree of dislocation entanglement (Figure 12(c1)) and deformation twins are formed in aluminum alloys (Figure 12(c2,c3)), which effectively impedes dislocation motion while also restoring work-hardening ability [148]. Dislocation stacking and twin-crystal stacking contribute to the increase in the rate of work hardening [149,150,151,152]. The nanoscale twin boundaries were reported to be capable of enhancing the capacity for dislocation accumulation, thereby contributing to improved plasticity [153,154]. Moreover, twin boundaries may serve as storage sites for dislocations, and longer and thinner ligamentous fossae exhibited superior material plasticity effects [155].

**Figure 13:**
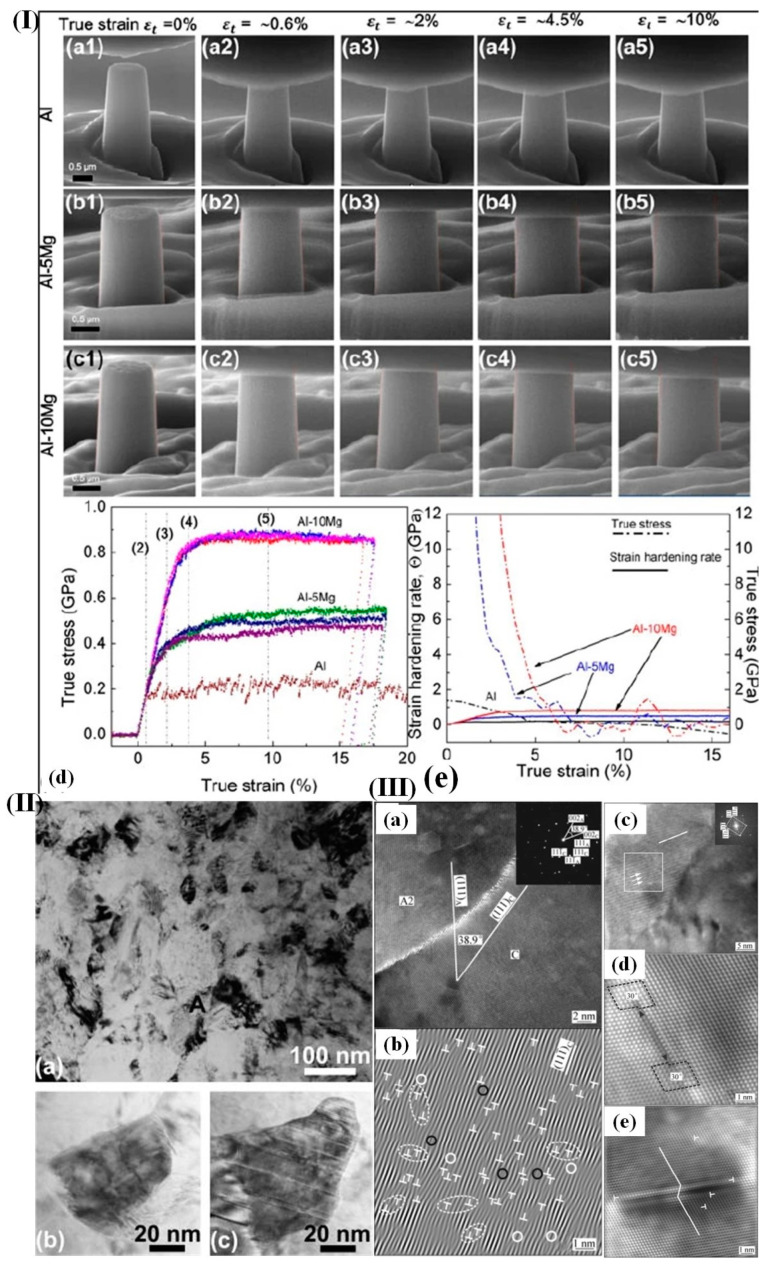
(**I**) (**a1**–**c5**) Scanning electron microscopy images depict the progression of these tests; (**d**,**e**) the true stress–strain relationship derived from an in situ compression test reveals a flow stress value close to 800 MPa specifically for the Al-10Mg thin film [158]; (**II**) (**a**) a representative bright-field TEM image of the HPT sample reveals a grain size range of 50–200 nm; (**b**) a typical image showcases a grain devoid of any twin or stacking fault; (**c**) another typical image illustrates a grain characterized by a high density of nanotwins and stacking faults [157]; (**III**) HRTEM images reveal a significant abundance of micro-twins and stacking faults (SFs) arising from the emission of partial dislocations by sub-boundaries and (**a**) the formation of a stacking fault through the dissociation of two 30° Shockley partials originating from an end-on 0° screw dislocation and (**b**) the occurrence of a four-layer twin resulting from the dynamic overlapping of four SF ribbons (**c**–**e**) [159].

Liao et al. [156] studied the dislocation densities in grains with and without twins/SFs based on high-resolution TEM images [157] (Figure 13II).

It was found that the presence of twins/SFs significantly increased the dislocation density up to approximately 2–5 × 10^16^ m^−2^, which was several times higher compared to grains without any twins/SFs (~0.5 × 10^16^ m^−2^). This observation strongly indicates that twins and SFs serve as effective barriers for slip propagation and act as storage sites for dislocations.

Xue et al. [158] prepared the epitaxial co-sputtered Al-Mg thin films with high-density growth twins. As shown in Figure 13I, the work-hardening rates of Al-5Mg and Al-10Mg are greater than that of pure aluminum. The AA5182 alloy [159] has extremely high dislocation densities near the nonequilibrium high-angle grain boundaries (HAGB) under the HPT process, as depicted in Figure 13III(b). As can be seen, the localized dislocation density reaches a remarkable value of 3.8 × 10^17^ m^−2^, which significantly surpasses the average dislocation density (1.3 × 10^15^ m^−2^) determined through X-ray profiling of the same sample [160]. Representative HRTEM images of microtwins and stacking faults (SFs) in subgrain A1 of the AA5182 alloy prepared under the HPT process are presented in Figure 13III(c–e) [161]. Specifically, Figure 13III(e) shows how a deformed twin crystal with a thickness of four atomic planes (~1 nm) is formed [162].

Furthermore, it is reported that grain boundaries play a pivotal role in the formation of twinned crystals. For instance, when the grain size is below 20 nm, over 10% of the atoms reside on grain boundaries [163], which contributes to the generation of laminar dislocations [164]. Twinned grain boundaries (TBs) are deemed as an optimal means to achieve a synergistic effect between superior ductility and enhanced strength compared to conventional high-energy GBs [17,165,166].

**Figure 14:**
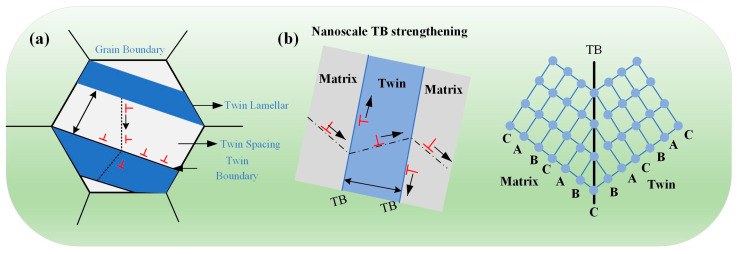
(**a**) The twinning deformation structure of the micro-nanomaterials; (**b**) enhanced strength at the nanoscale through interactions between dislocations and twin boundaries.

The twinning deformation structure of the micro-nanomaterials is shown in Figure 14a.

The stress concentration induced by the leading dislocations at the twin boundary is directly proportional to both the applied shear stress and the accumulated number of dislocations. As the thickness of the twinned wafer layer decreases, fewer dislocations can be trapped within the twin boundaries, resulting in an increased requirement for applied stress to drive dislocations across these boundaries. When the twinned layer becomes so thin that dislocation tucking is no longer feasible, an exceedingly high applied stress is necessary to facilitate single-dislocation crossing [167]. Consequently, the nanotwinned material mechanical properties are strongly influenced by the interaction between dislocations and twins with coherent twin boundaries because twin boundaries not only impede dislocation motion but also serve as sinks for absorbing dislocations under large plastic deformation.

## 5. The Effects of Diffusely Distributed Nanoparticles on the Microstructure and Properties of Al-Mg Alloys and Their Mechanisms

Particle-reinforced aluminum matrix composites have attracted widespread attention because of their low density, low coefficient of thermal expansion, high strength, and high thermal conductivity. The mechanical properties of the composites depend on the types of aluminum matrix (pure aluminum and aluminum alloy), the type of reinforcement (hard metal particles, carbon nanotubes, carbides, oxides, and nitrides). Hard metal particles are composed of Gr (graphite), Co (cobalt), Mn (manganese), W (tungsten).

The interface between the aluminum matrix and reinforcing phase particles plays a crucial role in determining the microstructure and mechanical properties of aluminum matrix composites. Interfacial bonding includes various interaction mechanisms, including mechanical, physical, and chemical bonding, directly influencing load transfer efficiency, material strengthening mechanisms, and fracture processes in composites. Mechanical bonding involves low interfacial energy due to friction between the matrix and reinforcing phase without dissolution or reaction. Physical bonding relies on physical or chemical adsorption with partial dissolution but no reaction between the matrix and reinforcing phase. Chemical bonding occurs when a new physical phase forms through reactions between the matrix and reinforcing phase. The moderate interfacial reaction is beneficial to bond formation while excessive interfacial bonding is detrimental.

The microstructure and mechanical properties of particle-reinforced aluminum matrix composites are closely related to their preparation process. The processing of the composites can be classified into four categories: solid-state methods (such as powder metallurgy and jet deposition), liquid-state methods (including stir casting, pressure infiltration, and vacuum suction casting), gaseous methods, and other advanced technologies (like in situ generation and additive manufacturing). Among these techniques, stir casting, pressure infiltration, in situ generation, and powder metallurgy are commonly employed. The mechanical properties of particle-reinforced aluminum matrix composites are influenced by various factors, including the type of aluminum matrix, the interfacial bonding between the matrix and reinforcing phase, as well as the characteristics pertaining to the reinforcing-phase particles such as their type, size, content, and dispersion within the matrix. Among these factors, particular emphasis should be placed on optimizing the size, content, and dispersion of reinforcing-phase particles in order to enhance the overall mechanical performance of these composites.

The extensive contact area between micron particles and the substrate causes inadequate interfacial bonding, leading to limited enhancement in strength. Consequently, the micron particles are prone to debonding and self-fracture under under loading, hindering material plasticity improvement. In contrast, aluminum matrix composites reinforced by nanoparticles with sizes less than one hundred nanometers exhibit superior interfacial bonding and enhanced resistance against particle breakage. Consequently, these composites exhibit excellent strength–plasticity synergy [168,169].

**Figure 15:**
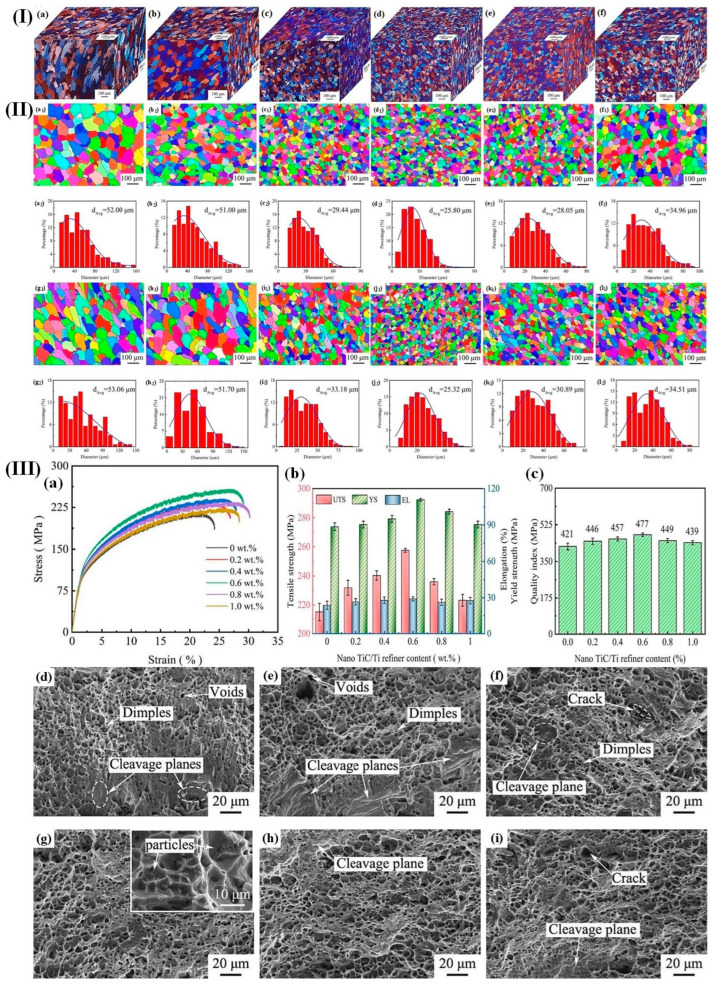
(**I**) (**a**–**f**) Microscopic structures of Al-Mg alloys containing varying weight percentages of nano-TiC/Ti grain refiner in three dimensions; (**II**) (**a1**–**f1**,**a2**–**f2**) EBSD mappings were conducted to analyze the distribution of grain and crystal orientation in Al-Mg alloys (yz plane) with varying weight percentages of nano-TiC/Ti grain refiner; (**g1**–**l1**,**g2**–**l2**) yz plane; (**III**) (**a**) stress–strain curves of Al-Mg alloy specimens containing varying weight percentages of nano-TiC/Ti grain refiner were analyzed; (**b**,**c**) the effect of nano-TiC/Ti grain refiner on the mechanical properties of Al-Mg alloys with varying weight percentages; (**d**–**i**) reconfiguration patterns of Al-Mg alloys featuring varying weight percentages of nano-TiC/Ti grain refiner [170].

Many investigations have found that the TiC particles can act as effective heterogeneous nucleation sites in Al alloys, leading to grain refinement. For example, Wang et al. [170] successfully prepared Al-Mg alloys with a nano TiC/Ti grain refiner using cold-metal-transition wire arc additive manufacturing (CMT-WAAM). They found that the nano TiC/Ti grain refiners had a significant effect on the microstructural characteristics and mechanical properties of CMT-WAAM-prepared Al-Mg alloys. The shape of the grains changed from columnar grains into equiaxed grains, accompanied by a refinement in grain size. The TiC particles served as effective heterogeneous nucleation sites, thereby enhancing the nucleation rate. Furthermore, TiC particles located at grain boundaries exerted an inhibitory effect on their growth. Figure 15III(a–c) reveals that the mechanical properties of Al-Mg alloys produced by CMT-WAAM were enhanced, with a microhardness of 91.18 HV, a yield strength of 110.80 MPa, a tensile strength of 257.60 MPa, and an elongation of 25.2%, which are improvements of 9.5%, 25%, 20%, and by 22%, respectively.

Specifically, the Al-Mg alloys containing a nano TiC/Ti grain refiner at a concentration of 0.6 wt% exhibited the maximum width and depth of craters (Figure 15III(g)), indicating superior toughness.

**Figure 16:**
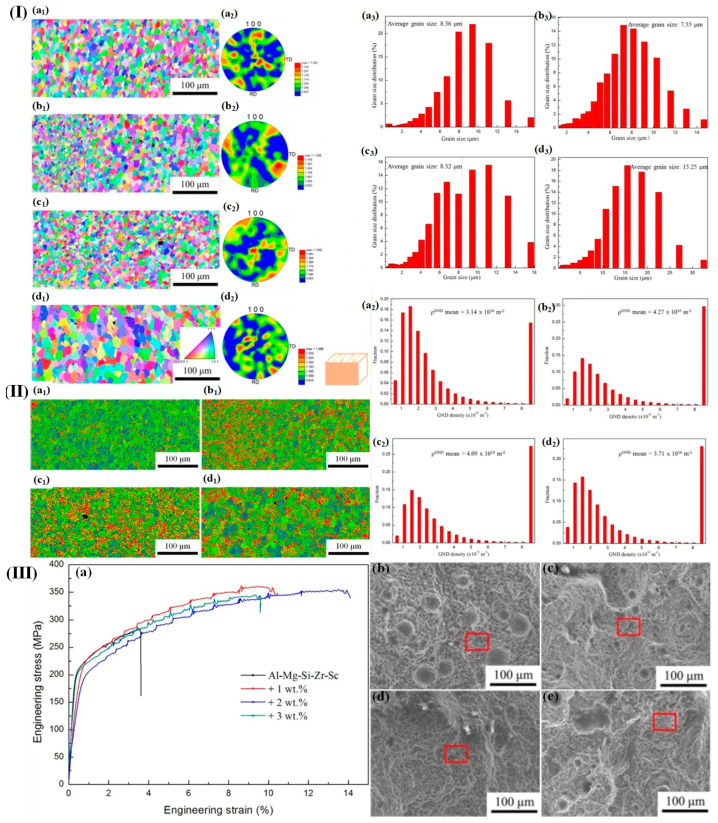
(**I**) (**a1**–**d1**,**a2**–**d2**) EBSD findings of TiC/Al–Mg–Sc–Zr alloys fabricated via DED; (**a3**–**d3**) size distributions of titanium carbide/aluminum–magnesium–scandium–zirconium alloys; (**II**) (**a1**–**d1**) variations in TiC content influence the KAM distributions of samples composed of Al–Mg–Si–Sc–Zr with different amounts of TiC; (**a2**–**d2**) density distributions of TiC/Al–Mg–Si–Sc–Zr samples with varying levels of TiC were computed. (**III**) (**a**) Room temperature engineering stress–strain behavior of TiC/Al-Mg-Sc-Zr composites; (**b**–**e**) scanning electron microscopy (SEM) images depicting the morphologies of fracture surfaces in Al-Mg-Sc-Zr alloys with varying levels of TiC [171].

Xu et al. [171] fabricated TiC-modified Al-Mg-Sc-Zr composites through direct energy deposition (DED) and investigated the influence of TiC ceramic particle content on their microstructure and properties. It is evident that a 1 wt% addition of TiC exhibits a pronounced grain refinement effect. With increasing TiC content, the refinement effect reduces because of the decrease in the number of heterogeneous nucleation sites and the coarsening of the TiC/Al-Mg-Sc-Zr grains (Figure 16I).

In addition, the incorporation of TiC contributes to an enhancement in the dislocation density of the aluminum alloy. Specifically, a significant increase in dislocation density (4.27 × 10^16^ m^−2^–4.09 × 10^16^ m^−2^) is observed for TiC contents ranging from 1 to 2 wt%, whereas a comparatively smaller effect is observed for a TiC content of 3 wt% (Figure 16II). The tensile properties of TiC/Al-Mg-Sc-Zr alloys were significantly enhanced and the incorporation of 1–2wt% TiC resulted in the optimal tensile properties (Figure 16III(a)). Specifically, the tensile strength increased from 283.25 MPa to 361.51 MPa, and the elongation increased from 3.61% to 14.10%. The fracture surface exhibited a ductile fracture mode with numerous fine equiaxial pits present. Moreover, in comparison to the TiC-free specimen, the abundant pores on its fracture surface potentially contribute to its low strength–plasticity behavior.

**Figure 17:**
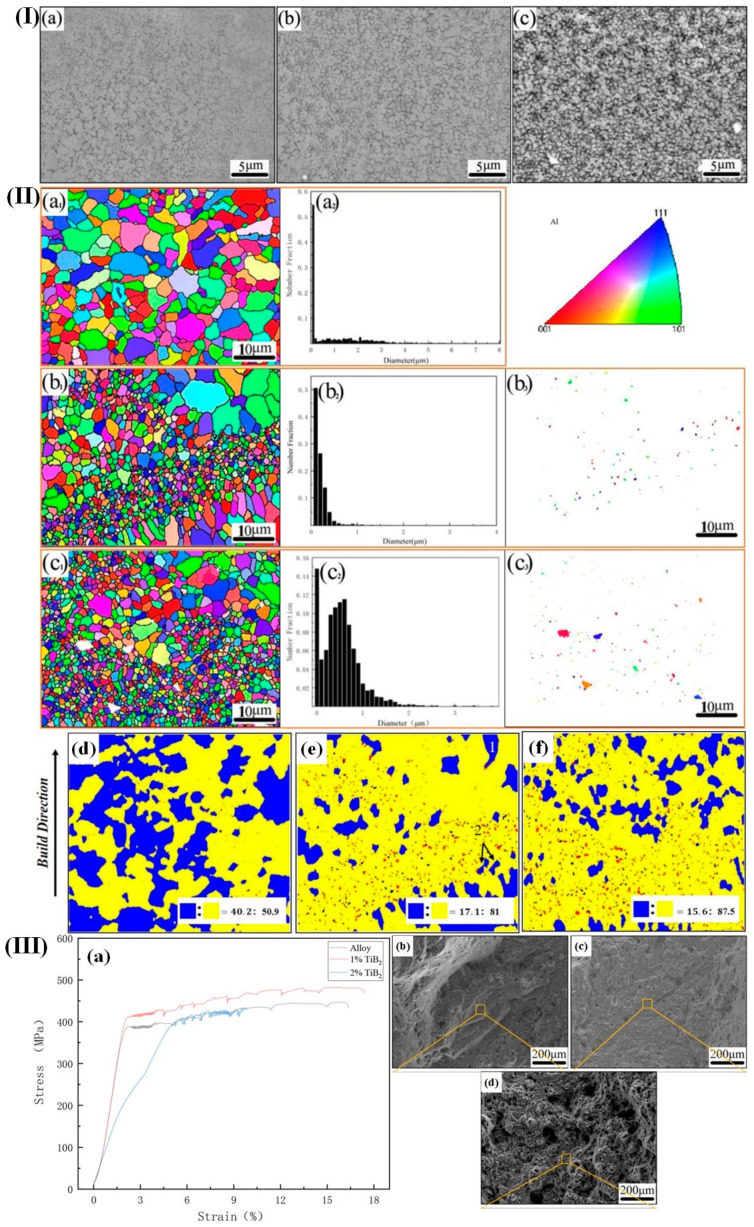
(**I**) (**a**–**c**) SEM-SE images depicting the X–Y plane in TiB_2_/Al-Mg-Sc alloys fabricated using SLM technology; (**II**) (**a1**–**c1**,**a2**–**c2**,**b3**,**c3**) electron backscatter diffraction images captured from the X–Y plane in alloys of TiB_2_/Al-Mg-Sc fabricated using selective laser melting; (**d**–**f**) fraction of recrystallization in the X–Y plane of TiB_2_/Al–Mg–Sc alloys fabricated using SLM technology; (**III**) (**a**) stress–strain behavior at ambient conditions of TiB_2_/Al-Mg-Sc alloys fabricated using selective laser melting; (**b**–**d**) fracture patterns observed in TiB_2_/Al–Mg–Sc alloys fabricated using selective laser melting [172].

Wu et al. [172] investigated the impact of TB2 addition on the microstructure and mechanical properties of Al-Mg-Sc alloys fabricated through selective laser melting (SLM). They found that an increase in TiB_2_ content leads to an increase in the width of the melt pool and a slight decrease in the depth of the melt pool, which may be attributed to the higher laser absorption exhibited by TiB_2_/Al-Mg-Sc alloys compared to Al-Mg-Sc alloys under identical process parameters. The microstructures of TiB_2_Al-Mg-Sc alloys fabricated by selective laser melting (SLM) comprised fine-grained, coarse-grained, and columnar crystalline structures (Figure 17II).

Notably, the proportion of fine-grained regions increases with increasing TiB_2_ content. Additionally, the introduction of TiB_2_ particles, which act as a heterogeneous nucleation agent for grain refinement and pinning sites for boundaries, drastically impedes recrystallization and grain growth during SLM. The TiB_2_/Al-Mg-Sc alloy with 1% TiB_2_ additions exhibited exceptional overall mechanical properties, with a tensile strength of 483 MPa and an elongation of 15.8%. In comparison to the Al-Mg-Sc alloy without TiB_2_ additions, the inclusion of 1% TiB_2_ in the alloy resulted in a notable increase in tensile strength of 37 MPa and elongation of 2.1% (Figure 17III(a)).

**Figure 18:**
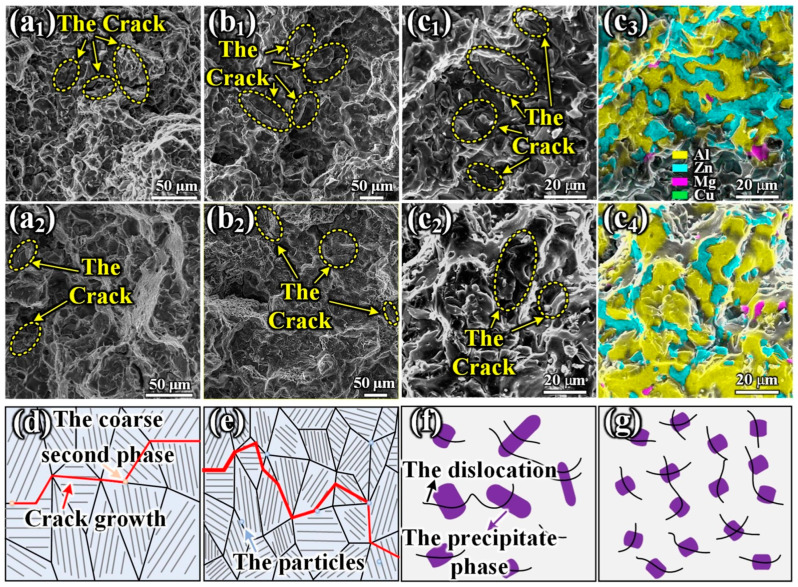
(**a1**–**c1**,**a2**–**c2**) Pattern of surface fractures; (**c1**,**c2**) EDS mapping analysis images are depicted in (**c3**,**c4**); (**d**,**e**) illustrated mechanism of enhancing toughness; (**f**,**g**) depicted interplay between dislocations and precipitates [173].

The mechanisms by which TiB_2_ + TiC particles enhanced the elongation and toughness of the alloy could be categorized into four aspects [173]. First, the grain refinement was beneficial for coordinated and uniform deformation between the grains, which reduced stress concentration and inhibited crack initiation. Additionally, an increased number of grain boundaries results in a more tortuous crack propagation path and greater consumption of elastic strain energy within the system (Figure 18d,e).

Secondly, TiB_2_ + TiC particles reduced the quantity of SPPs in the T6 heat-treated alloy, thereby reducing dislocation blockage and inhibiting crack initiation. Thirdly, the incorporation of TiB_2_ + TiC particles elevates both dislocation density and precipitate amount within the alloy. Compared to alloys, the alloy with the addition of TiB_2_ + TiC particles exhibited a twice-as-high dislocation density along with larger quantities of finer precipitates that are uniformly distributed (Figure 18f,g). These characteristics are responsible for the enhanced work-hardening behavior as well as the more uniform deformation due to the dislocation interaction, diffusion, and accumulation, resulting in ultimately improved elongation and toughness properties for these alloys. Finally, particle distribution hinders crack propagation across grain boundaries by deflecting cracks in various directions while consuming additional energy, thus augmenting toughness.

## 6. Summary and Prospects

The megaplastic deformation method for preparing ultrafine-grain aluminum and aluminum alloy materials has extended the application scope of traditional plasticity processing technology, leading to a significant improvement in their performance as well as the development of new materials. This advancement contributes to the enhancement of material service life, resource efficiency, waste reduction, and environmental protection, aiming to achieve sustainable development. Much progress has been made in ultrafine crystal nanocrystalline Al-Mg aluminum alloys processed by large plastic deformation. Specifically, severe plastic deformation (SPD) methods are becoming popular due to their capability for the refinement of the grain size of Al-Mg aluminum alloys to the submicron or nanoscale levels. Nanostructured materials possess superior mechanical properties due to their unique nanocrystalline structure compared to coarse crystalline metals, so they are very attractive for various structural and functional applications. However, it should be noted that nanocrystalline materials may exhibit significantly higher strength than that of coarse crystalline metals; they also experience a certain decline in ductility due to the reduced work-hardening ability.

Various strategies have been proposed to enhance the plastic deformation behavior of ultrafine/nanocrystalline materials. These methods include incorporating bimodal structured materials, nanotwins, or diffusely distributed nanoparticles to the alloys. They are primarily focused on restoring and maintaining the work-hardening capability of nanostructured materials through tailored modifications aimed at improving dislocation accumulation ability.

The technology of megaplastic deformation for the preparation of nanograin materials has been gaining increasing attention. However, in order to widely apply this technology to practical industry application, further comprehensive research is necessary. First, determining reasonable parameters and optimizing the process route for large plastic deformation can maximize the uniformity, continuity, and overall mechanical properties of ultrafine-grain aluminum and aluminum alloy materials. Secondly, new technologies and methods that are suitable for the efficient, continuous, and rapid industrial production of large plastic deformation-prepared ultrafine-grain aluminum and aluminum alloys will facilitate a technological shift towards the industrial production of high-volume materials. Above all, there is a lack of research on the toughening mechanism compared to the strengthening mechanism in large plastic deformation ultrafine-grain nanocrystalline Al-Mg aluminum alloys. Therefore, further in-depth studies on the strengthening and toughening mechanisms in large plastic deformation aluminum alloys are needed to develop ultrafine crystalline and nanocrystalline Al-Mg aluminum alloys with high strength and toughness.

## Data Availability

Not applicable.
